# Ensuring Optimal Outcomes for Preterm Infants after NICU Discharge: A Life Course Health Development Approach to High-Risk Infant Follow-Up

**DOI:** 10.3390/children11020146

**Published:** 2024-01-24

**Authors:** Jonathan S. Litt, Neal Halfon, Michael E. Msall, Shirley Ann Russ, Susan R. Hintz

**Affiliations:** 1 Division of Newborn Medicine, Department of Pediatrics, Harvard Medical School, Boston, MA 02115, USA; 2 Department of Social and Behavioral Pediatrics, Harvard TH Chan School of Public Health, Boston, MA 02115, USA; 3 Center for Healthier Children, Families, and Communities, University of California, Los Angeles, CA 90024, USA; nhalfon@mednet.ucla.edu (N.H.); sruss@mednet.ucla.edu (S.A.R.); 4 Department of Pediatrics, University of California, Los Angeles David Geffen School of Medicine, Los Angeles, CA 90024, USA; 5 Department of Health Policy and Management, UCLA Fielding School of Public Health, Los Angeles, CA 90095, USA; 6 Department of Public Policy, UCLA Luskin School of Public Affairs, Los Angeles, CA 90095, USA; 7 Department of Pediatrics, Sections of Developmental and Behavioral Pediatrics and Kennedy Research Center on Intellectual and Neurodevelopmental Disabilities, University of Chicago Medicine, Chicago, IL 60637, USA; mmsall@bsd.uchicago.edu; 8 Division of Neonatal and Developmental Medicine, Department of Pediatrics, Stanford University School of Medicine, Palo Alto, CA 94305, USA; srhintz@stanford.edu

**Keywords:** life course health development, preterm birth, high-risk infant follow-up, long-term outcomes, thriving, flourishing, neurodiversity

## Abstract

Children born prematurely (<37 weeks’ gestation) have an increased risk for chronic health problems and developmental challenges compared to their term-born peers. The threats to health and development posed by prematurity, the unintended effects of life-sustaining neonatal intensive care, the associated neonatal morbidities, and the profound stressors to families affect well-being during infancy, childhood, adolescence, and beyond. Specialized clinical programs provide medical and developmental follow-up care for preterm infants after hospital discharge. High-risk infant follow-up, like most post-discharge health services, has many shortcomings, including unclear goals, inadequate support for infants, parents, and families, fragmented service provisions, poor coordination among providers, and an artificially foreshortened time horizon. There are well-documented inequities in care access and delivery. We propose applying a life course health development framework to clinical follow-up for children born prematurely that is contextually appropriate, developmentally responsive, and equitably deployed. The concepts of health development, unfolding, complexity, timing, plasticity, thriving, and harmony can be mapped to key components of follow-up care delivery to address pressing health challenges. This new approach envisions a more effective version of clinical follow-up to support the best possible functional outcomes and the opportunity for every premature infant to thrive within their family and community environments over their life course.

## 1. Introduction

Infants born preterm (<37 weeks’ gestation) are at risk for health and developmental challenges in childhood and beyond when compared to term-born peers [1]. These problems include chronic diseases of the respiratory, cardiovascular, and renal systems, neurosensory impairments, and delays in motor, language, and social development [2,3]. This risk increases as gestational age at birth decreases [4]. Despite increasing survival for infants born at the earliest gestations, rates of neurodevelopmental impairment have not similarly improved [5]. Children born preterm have high rates of rehospitalization after discharge from the neonatal intensive care unit (NICU) [6] and require considerable medical services and developmental support as a consequence of their ongoing needs [7]. Noting the significant impact of preterm birth on physical health and neurodevelopment, and the longitudinal needs for medical and therapeutic services, organizations from several industrialized countries have offered guidance for clinical HRIF programs. These efforts—sharing the best intentions to provide standard guidance for variable and often inequitable care—differ in levels of stakeholder representation, specificity, and actionability. In 2022, the Canadian Paediatric Society crafted a position statement on preterm infant follow-up in Canada, including broad recommendations for medical, nutritional, and developmental surveillance and support [8]. In the United Kingdom, the National Institute for Health and Care Excellence published a guideline for the developmental follow-up of preterm children in 2017. Recommendations include information and support to parents and caregivers prior to NICU discharge, enhanced surveillance for developmental challenges in early childhood, and referrals to appropriate community-based therapeutic services [9]. And the Centre of Research Excellence in Newborn Medicine is currently creating a practice guideline for very preterm infant follow-up in Australia [10].

In the United States, the American Academy of Pediatrics recommends that all ‘high-risk’ infants receive medical and developmental follow-up care after NICU discharge [11,12]. Though sharing some similarities in structure, content, and timing, this specialized clinical follow-up provides a set of health services distinct from those typically used to collect outcome data solely or primarily for research purposes or NICU quality improvement initiatives [13]. Although considered the standard of care, the structure, content, and timing of high-risk infant follow-up programs in the US are highly variable [14]. Participation in follow-up programs for high-risk neonates is a required component of accredited neonatal-perinatal medicine training programs [15]; however, there is no uniform or standardized curriculum guiding training activities across institutions. Given that preterm birth and early-life morbidity have a profound impact on health and development over the life course, infants and their families face logistical, financial, and emotional challenges in the transition from the NICU to the home and community that the existing system of follow-up is ill-equipped to address. The fractured US healthcare environment is often unable to meet these often highly vulnerable infants’ and families’ complex needs. Here we propose a life course health development approach to clinical follow-up for preterm infants that holds the potential to transform follow-through to ensure optimal trajectories of health, development, and thriving in a coordinated proactive family and community support network.

## 2. Overview of Clinical High-Risk Infant Follow-Up Care

Community-based primary care pediatricians provide the majority of ongoing care to infants and families after NICU discharge and serve as the key source of longitudinal general pediatric care [16,17], ideally delivered in the setting of the family-centered medical home [18,19]. Formal follow-up programs providing specialized medical and developmental surveillance and support for preterm and other high-risk infants occur separately from primary care practices in a number of designated settings, including academic medical centers, community-based clinics, or, in some instances, even the infant’s home. Training in follow-up care is considered a core competency for neonatal-perinatal medicine fellowship programs and is a requirement for training program accreditation, though specialized follow-up may also be provided by developmental pediatricians, neurologists, or complex care programs.

The scope and focus of follow-ups have yet to be standardized and often vary among programs. Some programs provide neurodevelopmental support alone, while others offer more comprehensive medical services, even integrating well and sick visits with developmental assessments and subspecialty care [14,20]. Multidisciplinary follow-up programs are common and include physicians, physical and occupational therapists, speech-language pathologists, dietitians, social workers, psychologists, and care coordinators as members of the care team in providing support to infants and families [21,22,23,24]. While most follow-up care is provided in the clinic setting, a novel transition to home programs has successfully integrated home visits after NICU discharge [25,26]. Many programs began using telehealth platforms for follow-up care during the COVID-19 pandemic [27,28]. A recent systematic review found that care coordination for infants with complex care needs reduces costs for families and health systems and leads to improved care quality [29].

There is no standard guiding the optimal timing of clinical follow-up visits. Some programs have an early visit to help the family with the transition home. More commonly, programs offer visits to eligible infants at regular intervals: 3–6 months, 8–12 months, 18–24 months, and 30–36 months corrected age [12,14]. This cadence of visits has roots in clinical research studies in the NICU that often use a predetermined sequence of developmental assessments at these ages as the standard measure of primary or secondary outcomes. Clinical follow-up programs typically end at 2 or 3 years, with some extending into early school ages (5–8 years). Alternatively, the frequency of visits may be dictated by medical or developmental needs rather than strict age ranges. For example, a novel Canadian follow-up program focuses on specific developmental “touchpoints” for the child and family to support the unique needs of each developmental stage [30]. This type of approach is more reflective of, and responsive to, infant and family needs as they evolve over the whole of childhood and promotes collaboration with developmental, educational, and behavioral systems of care.

## 3. Challenges, Obstacles, and Shortcomings

There is a myriad of material, logistical, and philosophical challenges to providing high-quality, equitable follow-up care after NICU discharge so that children optimize their physical and behavioral health and thrive in school and the community throughout childhood and adolescence. Foremost is an overall lack of clear goals, mission, and vision for clinical follow-up for preterm infants. Programs are largely based on a medical model that focuses on the child alone without explicit attention to the developmental context in which he or she lives. Absent the moral urgency to, in the words of Jeffrey Horbar [31], “follow-through” on the investments made in the NICU after discharge and beyond, centers are left to make individual determinations about the scope and resources allocated to medical and developmental supports locally.

Next, there is significant variability in the availability and utilization of follow-up services among programs, centers, and regions, due in large part to a fractured healthcare system, with multiple siloed services and programs. The likelihood of missing needed services and experiencing unmet care needs is all too common. For example, nearly 30% of extremely low birth weight infants are rehospitalized in the first two years of life. Nearly 50% miss recommended neurosensory screening [32]. Children born preterm are less likely to have a family-centered medical home compared to term-born peers [33]. There are frequent barriers to early intervention service receipt [34] and many have missing or delayed childhood vaccinations [35]. Minority maternal race/ethnicity status and higher infant gestational age or birthweight are associated with missed referrals. Because of differences in personnel, resources, and approaches among HRIF clinics as well as variations in NICU discharge planning, there are substantial gaps in implementing the best preventive, coordinated, and proactive support for the family and the developmental care team. Other barriers include dissemination of the knowledge of HRIF eligibility criteria and referral processes that influence referral and follow-up rates [14,20,36].

Last, there are additional obstacles to HRIF posed by structural and interpersonal racism, classism, anti-immigrant bias, and financial stress that compound the burdens experienced by preterm infants and their families. There are marked racial inequities between hospitals in the rates of follow-up [37]. A study from the California Perinatal Quality Care Collaborative found that infants born to Black (OR 0.58, 0.47–0.71) and Hispanic (OR 0.65, 0.55–0.76) mothers compared to white were less likely to be referred to follow-up programs [38]. Infants born to a Black mother, having a family with limited English proficiency, or residing in a neighborhood with very low economic and educational opportunities decrease the likelihood of HRIF participation [39]. This is also true of having public insurance, living in a rural area, or residing at a significant distance from the follow-up location [40]. Parents report that travel logistics and costs prohibit adhering to recommended follow-up [41,42,43]. Limited clinic hours and conflicts with parent work schedules posed additional barriers to participation [44]. Having an initial follow-up visit increases the likelihood of attending future visits [45], highlighting the importance of addressing inequities in the discharge preparation processes and transition to home.

In sum, a preponderance of evidence indicates that much of the routine clinical follow-up may constitute low-value, low-quality care, given the high degree of systematic fragmentation, incompleteness or redundancy of services, and inequity in distribution. This is not due to the lack of commitment, passion, knowledge, or good will from those dedicated clinicians providing follow-up care in both academic and community settings. Rather, to the contrary, it is a failure of care systems, with a lack of a clear and unified vision, chronic underinvestment in human capital and material resources, and constraints imposed by quixotic health and social policy landscapes. To chart a new course, we propose using the life course health development framework to ground a coherent system of follow-up care for preterm infants beyond the first 2 years of life. Ahead, we define the seven principles of life course health development and map each to critical aspects of HRIF. In this way, we offer a theory-based approach to designing follow-up systems that are developmentally responsive, family-centered, contextually appropriate, and enablement-informed.

## 4. Life Course Health Development

**Figure 1:**
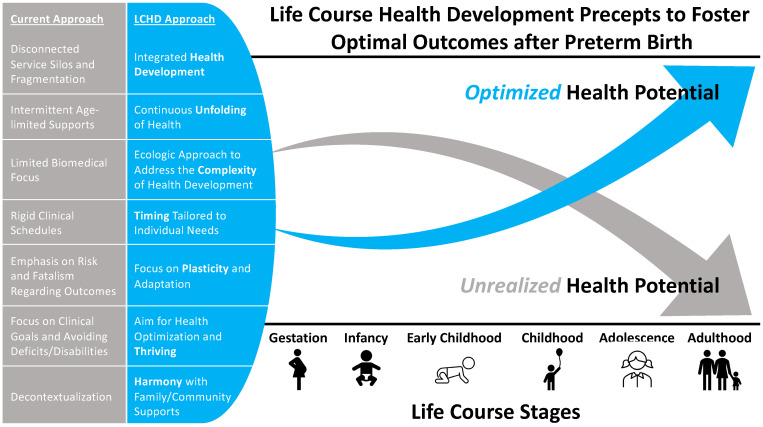
The life course health development (LCHD) approach to optimizing health and development for preterm infants and families after NICU discharge. The current approach (gray box) creates and perpetuates barriers to achieving optimal health potential. The proposed LCHD approach (blue box) guides the building of care models and integrated health, education, and social support systems to foster positive trajectories of health potential over the life course. Bolded words represent each of the seven LCHD principles.

The life course health development (LCHD) approach regards health not as a static phenomenon but as a dynamic, emergent capacity that develops continuously over the lifespan through a complex non-linear process [46]. This process can be represented as a health development trajectory that changes over time in response to multiple risk and protective factors transmitted in a relational developmental matrix, designated the child’s developmental ecosystem. These risk and protective factors can have an “outsize” influence during critical and sensitive developmental periods, many of which occur at the start of life, when biological and behavioral regulatory systems are being programmed [46,47]. In 2018, Drs. Neal Halfon and Christopher Forrest proposed the seven principles of life course health development. Drawing from foundational, empirically grounded work in life course epidemiology [48], biologic and social ecology [49], and the developmental origins of health and disease [50] among other theories and frameworks, the LCHD perspective incorporates fundamental questions about biological and behavioral plasticity and adaptation and how interactions between biology and environmental contexts lead to disease or health in individuals and populations [51]. Since its publication, the LCHD framework has been applied by numerous investigators in fields as varied as congenital heart disease [52], neuropsychiatric disorders [53,54], and healthcare delivery [55,56]. Msall et al. applied this framework in identifying research strategies to inform evidence-based interventions for “optimizing physical and behavioral health, educational achievement, and adaptive competencies” among preterm infants [57]. In this paper, we employ the seven principles of the LCHD framework (see Table 1, Figure 1) to highlight the core functions of follow-up care for infants born preterm and, in so doing, propose a series of transformative changes to the system by which we might achieve optimal health development outcomes for this vulnerable population.

I. Health Development. Fundamental to the LCHD framework, the concept of health development integrates the notion of health, as defined as a set of attributes “…that are desirable, acquired, optimized, and maintained during the life course, enabling growth of an individual, survival, and adaptation to manifold environments” and the active process of development by which health attributes change over time [51]. The interdependence of health and development may seem obvious to parents, teachers, and pediatricians, yet the institutions and systems charged with studying disease mechanisms, developing health interventions, and implementing evidence-based solutions treat health and development as distinct entities. This is exemplified by increasingly deep yet narrow biomedical research interests and ever-disconnected organ-based clinical subspecialties.

For children born preterm, the schism between health and development obscures a comprehensive understanding of the impact of prematurity on well-being over time and limits the scope of potential interventions to improve functional outcomes. The multimorbid conditions for which preterm infants are at risk in the neonatal period—bronchopulmonary dysplasia, necrotizing enterocolitis, and intraventricular hemorrhage to name but a few—have common and well-documented associations with neurocognitive development in early childhood [58,59]. Related chronic health problems, especially those affecting lung function, are associated with inattention [60], executive function challenges [61], dysregulated mood and behavior [62], and suboptimal academic achievement [63]. With few exceptions, investigators treat static diagnoses of biological health in the perinatal period as individual threats to development in later life. Such cross-sectional approaches neglect the co-occurring and likely interacting trajectories of physical symptoms, behavior, and developmental functional skills. This is reflected by the typical approach to clinical follow-up care that silos organ systems with disease- or symptom-specific assessments and therapies isolated in time and space. A more dynamic approach would not only give insight into the underlying mechanisms and pathways by which preterm birth leads to adverse health, developmental, and educational outcomes but also lead to potentially more effective holistic interventions to improve those outcomes. *To optimize health (state of being) and development (process), outcomes research and the follow-up care that ensues must be interdisciplinary, collaborative, and integrated among providers and across settings of health, education, and community support.*

II. Unfolding. Health development is a process that occurs continuously over one’s lifetime. Unfolding describes a dynamic and non-linear evolutionary process of development from simple to ever more complex forms and behaviors, shaped in response to experience and environmental interactions. Physiologic and behavioral adaptation to external stimuli is achieved through iterative loops of cellular and bodily sensing, signaling, and regulation (self-organization) [51] that are also the basis of maternal–child interactions, early child developmental competencies, and learning at school and with peers.

Preterm birth is a disruption of the typical unfolding process, leading to maturational immaturity that can have lifelong consequences for health development [64]. Studies have shown changes to telomeres and DNA methylation—markers of cellular age—associated with being born preterm [65,66]. NICU interventions and environments can exacerbate this underlying threat. The short-term impact on health development is often readily apparent, given the multi-organ morbidities and prolonged hospital stays associated with prematurity. The near-term effects on growth, and motor, cognitive, and language development, are well-documented. The long-term behavior, mood, and social relatedness problems are often latent, appearing only as the child grows to adolescence and adulthood. *Follow-up should reflect that the impact of prematurity is not limited to the perinatal period or even early childhood but, rather, constitutes a lifelong interrelated cascade of vulnerabilities that pose ongoing challenges to the typical processes of developmental unfolding and opportunities for resiliency. Medical care and other support services must provide the anticipatory foresight necessary to consider a longitudinal approach starting in the antepartum period and spanning childhood and beyond. This must include population data for kindergarten readiness, school success in literacy and numeracy, and adolescent efficacy in physical and behavioral health and community participation. They also must integrate emerging research on the effects that preterm birth, perinatal illness and treatments, and social and environmental stresses have on the epigenome and the physiologic processes it regulates.*

III. Complexity. Health development is an iterative, multilevel process of reciprocal interactions between individuals and their physical, natural, and social environments [51]. This concept has been articulated by Urie Bronfenbrenner in his bioecological theory, in which a child’s growth and development occur at the center of an ever-expanding ring of social contexts, from the nuclear family (microsystem) to community institutions (mesosystem) to local, state, and national policies (exosystem) to widespread cultural norms and ideologies (macrosystem), all unfolding over time (chronosystem) [67]. The pivotal role of the environment and context on health development is supported by robust and longstanding evidence from medical and developmental biology, public health, psychology, and education literature. Adverse childhood experiences (ACEs) that manifest and emerge out of complex family developmental ecosystems, which may include maltreatment, exposure to domestic violence, or untreated and unresolved mental health needs, are often associated with poor physical and mental health in adulthood [68]. Another complex emergent process is the phenomena known as toxic stress, i.e., the process by which early-life material deprivation and adversity, often embedded in a historically structured network of racist policies and processes, become embodied and expressed as poor physical, behavioral, and social health in adulthood [69]. Both seat health development in context, highlighting the need for multilevel interventions to abate adverse exposures and support resilience and growth. Effective, dynamic multilevel interventions that incorporate action on the social and structural determinants of health could prove transformative in supporting health equity from the start and in reducing or even eliminating longstanding inequalities in health across the lifespan [70].

The risk for preterm birth is not equitably distributed, with Black and poor communities at the highest risk [71]. This inequity is then reinforced by disproportionate rates of neonatal morbidities and associated chronic health problems related to prematurity [72]. Tragically, post-discharge services for NICU graduates such as early intervention [73] and high-risk infant follow-up programs [39,74] are also less available to Black, poor, and non-English speaking populations, further perpetuating poor health development outcomes. Fragmentation of health systems, social services, and education systems is common, leading to many and frequent unmet needs [33]. Administrative burdens and barriers in the systems intended to offer support to infants and families converge to drive individual outcomes. Yet interventions are often directed at individual patients and families, rather than the inadequate systems with which they interface. In other words, the existing approach to interventions is primarily driven by outdated biomedical models, with inadequate attention to the broader relational developmental ecosystems that are shaping children’s health development and sense of well-being [75]. *Interventions must be responsive to a child’s social and developmental context and multilevel in scope, not simply delivered to the individual infant or family, and include accessible pathways for developmental, behavioral, and educational services.*

IV. Timing. Developmental processes of any kind are by definition time time-delimited. The life course health development framework centralizes the role of timing of environmental exposures and experiences in guiding child well-being [51]. Fetal and neonatal life represent exquisitely sensitive periods of growth and development, particularly with respect to the nervous system [76]. Yet both vulnerability and heightened adaptability may persist through early childhood and beyond, during which time physical, environmental, and social exposures may threaten or support developmental trajectories [77]. A robust literature supports the importance and potential effectiveness of early interventions for children with medical and developmental complexity to achieve optimal functional outcomes [78].

Providers of neonatal intensive care are acutely aware of the impact of timing on health, where additional days or weeks of gestation may make a discernable difference in medical outcomes. Achieving physiologic stability during the ‘golden hour’ after delivery is understood to be critical to reducing morbidity during the NICU course [79]. A similar approach may be applied to follow-up after hospital discharge, in which high-risk preterm infants receive care and support that anticipates and addresses developmental problems as they emerge. Current models of follow-up are highly regimented and protocolized. An example of a more time-responsive and developmentally responsive approach to follow-up is Page Church’s touchpoints model, in which assessments and interventions are matched to the age-related developmental skills of each child and tiered to address the learning, behavioral, social, and habilitative supports in children at risk for a spectrum of neurodevelopmental challenges that unfold [30]. Traditional follow-up for preterm infants stops at preschool or an early school age, yet new problems with behavior, mood, and academic performance may arise at school ages and beyond [13]. Although it is clearly outside the purview of neonatal medicine to provide care at these ages, there is a collective responsibility to ensure that proactive collaborative systems exist for continued support throughout childhood, adolescence, and young adulthood. *Assessments and interventions need to be developmentally attuned with an eye toward continuously responsive follow-through, not administered according to predetermined timelines and age ranges.*

V. Plasticity. Living organisms adapt and evolve to their changing environments. Evolutionary theory posits that alterations in climate, the abundance or scarcity of food, and the presence or absence of predators act as selective pressures that lead to adaptation or extinction. Plasticity refers to the developmental capacity to “shift gears” to adapt to diverse and changing environments. For individuals, this occurs at the molecular and behavioral level. Yet societies and cultures can evidence adaptive strategies that help support the phases and life stages of health development [51].

Infants and young children display remarkable plasticity and adaptability in the face of early adversity (see #3 Complexity above). Preterm infants display a range of developmental and functional outcomes at an array of gestational ages and across postnatal developmental epochs. There is similar variability in outcomes among medical centers, states, and regions. The concept of reproductive casualty, the notion that a suite of certain fixed health and developmental outcomes are to be expected for any given gestation at birth, is not supported by evidence. On the contrary, timely antenatal and postnatal interventions, high-quality neonatal intensive care, access to needed supportive services, and, above all, enriched home and community environments can help buffer the developmental threats posed by preterm birth. Thanks to a high degree of physiologic plasticity, the gestational age at birth is indeed not destiny [80]. For this reason, *follow-up programs must have a strengths-based approach that harnesses all the potential factors in the family, community, and wider ecosystem that can be brought to bear on enhancing each child’s developmental progress. This approach benefits from an emphasis on individual goal setting, working toward the ‘possible,’ emphasizing supports for self-care independence, basic learning, social and community living skills, and avoiding prognostic certainty for long-term functional outcomes.*

VI. Thriving. Health development, and the environments and resources that optimally support and adaptively maintain it, “enable individuals to pursue desired goals and live long, flourishing lives.” [51]. In this sense, health development is not merely the absence of disease but rather a state of whole-self adaptive fulfillment. A thriving individual has the internal capacity and external resources that provide them with the resilience they need to avail themselves of all potential opportunities for contented living and optimize participation in health, home, community, and social activities.

For preterm infants, successful outcomes have historically been defined as a lack of medical morbidity and developmental delay as measured on standardized tests. Yet parents report functional abilities, participation with family and peers, and happiness to be of equal or greater value to medical and developmental diagnoses [81]. And adults born preterm themselves rate their own quality of life as good or very good [82]. It is vital to focus on achieving optimal health development for all children, not normalcy or perfection against common standards. Thriving may look different to different families. *Outcomes need to reflect family-identified values and definitions of thriving, for both the child and their family, and be informed by neurodiversity and enablement. In this context, there is a strengths-based approach that is not deficit remediation and cumulative diagnostic overshadowing and aligns with the International Classification of Functioning, Disability, and Health* [83]. *The ICF framework also aligns with the 6F-Words highlighted for partnerships with children and families. These enablement words include function by doing what you can, fitness for proactive health, fun by having a passion for the achievable, family and friends for support and encouragement, and a future of possibilities* [84].

VII. Harmony. Health development is the end of the result of several distinct and intersecting processes occurring across the life course [51]. Environmental forces act on the individual organism that has its own embodied molecular and behavioral response. For individuals and populations alike, there is a balance between vulnerability and resilience that leads to health and developmental outcomes [85].

Physical, cognitive, emotional, and social development require different developmental skills that all have different trajectories, with different time signatures, and that need to be aligned and harmonized over time for preterm infants to optimally thrive as they age [86]. Not only is that harmonization a biological necessity, but it is also a social, cultural, and psychological goal. Children, their siblings, and their parents adapt and grow at different paces. Cultural and political forces can derail health development—examples include racism, xenophobia, sexism and misogyny, heterosexism and homophobia, ableism, and poverty. Environmental exposures literally become ‘embodied’ through the allostatic load of toxic stressors [87]. Premature infants’ developmental trajectories across different domains may not follow expected norms and expected time frames. Follow-up services need to be deployed, understanding the dyssynchronous nature of these health developmental processes and finding the best ways to provide individualized anticipatory guidance and support to the entire family unit, acknowledging that developmental timings may not follow the same course as for term infants, even beyond early childhood. *Providers and health systems must work to dismantle organizational barriers, adopt more flexible timing for services and supports across the life course, and advocate for institutional changes and policy solutions to remedy past and present injustices. These changes can support families as they grow and facilitate optimal health development over the life course.*

## 5. Conclusions and Future Steps

True, lasting improvement to the current medical model of high-risk infant follow-up requires three key actions. First, clinicians and program leaders must engage with all stakeholders, starting with families, and including community-based pediatricians and family practice providers, physiatrists, therapists, educators, health systems, and policy-makers to co-design a new system of follow-up that is responsive to new knowledge from epigenetics and neurobiology about the nature of health development. Second, effective, high-quality, high-value follow-up care can only be achieved through a multilevel systems approach and not only through individual-level interventions. Third, there must be a commitment to equity at every step. The principles of life course health development offer a framework and set of principles to guide innovative, developmentally responsive, and context-specific care for preterm infants after NICzU discharge, providing follow-through on the commitments made to infants and their parents at the outset to achieve the best possible outcomes for health, development, functioning, participation, and fulfillment.

## Figures and Tables

**Table 1:** Life course health development principles applied to follow-up for preterm infants *.

Principle	Brief Description	Current Approach	New Approach
1. Health Development	Health development integrates the concepts of health and developmental processes into a unified whole	Medical and developmental care and support conceived as separate strands of care and provided by discrete practitioners in siloed settings	Child- and family-centered health development care provided by integrated teams utilizing tools to enhance shared goal-setting and inter-team communication
2. Unfolding	Health development unfolds continuously over the lifespan, from conception to death, and is shaped by prior experiences and environmental interactions	High-risk infant follow-up programs provide time-limited support to infants and families through age 2 or 3 years	Embracing foresight and anticipating future needs, systems of care must consider a longitudinal approach to supporting those affected by preterm birth starting in the antepartum period and spanning key transitions during childhood and beyond
3. Complexity	Health development results from adaptive, multilevel, and reciprocal interactions between individuals and their physical, natural, and social environments	Narrow, biomedical focus on clinical needs, often ignoring the direct impact of family and social context	Systems approach to understanding, assessing, and addressing multilevel drivers of health development, including social, structural, and developmental determinants Creation of tailored, agile, adaptive, multilevel interventions
4. Timing	Health development is sensitive to the timing and social structuring of environmental exposures and experiences	Developmental assessments performed according to a pre-determined timetable (e.g., 6, 12, 24 months of age), ignoring sensitive periods and transitions and turning points in developmental pathways	Follow-up visits are arranged as clinical needs arise; the timing and cadence of developmental assessments and interventions reflect the unfolding needs and skills of the child during sensitive periods of development
5. Plasticity	Health development phenotypes are systematically malleable and enabled and constrained by evolution to enhance adaptability to diverse environments	Medical language of ‘risk’ for ‘poor outcomes’ and ‘bad diagnoses’ communicates fatalism around the fixed impact of gestational age on health development	Language and practices to optimize resilience are incorporated into discussions of diagnoses, goal setting, and expected outcomes, emphasizing functioning and participation
6. Thriving	Optimal health development promotes survival, enhances well-being, protects against disease and promotes functioning in physical, behavioral, and social health	The structure, content, and cadence of follow-up visits determined by medical experts to serve pre-determined clinical goals. The focus is on diagnosing and managing impairments	Follow-up programs reflect the expressed needs and values of parents for their children with an emphasis on optimizing function and participation. The focus is on optimizing health development at all stages of life so that measurable successes occur
7. Harmony	Health development results from the balanced interactions of molecular, physiological, behavioral, cultural, and evolutionary processes	Follow-up focuses on de-contextualized individual deficits and offers medical solutions to socially mediated problems in siloed, fragmented settings	An integrated, systems- and strengths-based approach to supporting health development over the life course, utilizing family-generated assessments harmonized with their needs and goals and providing accessible medical, educational, and community supports

* Based on the original with permission of the authors. Halfon N, Forrest CB. The Emerging Theoretical Framework of Life Course Health Development. 21 November 2017. In: Halfon N, Forrest CB, Lerner RM, et al., editors. Handbook of Life Course Health Development [Internet]. Cham (CH): Springer; 2018. Available from: https://www.ncbi.nlm.nih.gov/books/NBK543722/ (accessed on 10 January 2023) doi: 10.1007/978-3-319-47143-3_2 [51].

## Data Availability

No new data were created or analyzed in this study. Data sharing is not applicable to this article.
